# Mapping the monoclonal antibody landscape of human cytomegalovirus

**DOI:** 10.1128/jvi.00325-26

**Published:** 2026-06-15

**Authors:** Maria K. McClave, Ekaterina E. Heldwein

**Affiliations:** 1 Department of Molecular Biology and Microbiology and Graduate Program in Molecular Microbiology, Graduate School of Biomedical Sciences, Tufts University School of Medicine 228275 , Boston, Massachusetts, USA; Universiteit Gent, Merelbeke, Belgium

**Keywords:** HCMV, monoclonal antibody, neutralization, effector functions, gB, gH/gL, pentamer, trimer, gM/gN, non-envelope antigens

## Abstract

Human cytomegalovirus (HCMV) is a major cause of congenital disease and a significant threat to immunocompromised individuals, particularly transplant recipients and neonates. Over the past five decades, numerous monoclonal antibodies (mAbs) targeting various HCMV antigens have been isolated. Here, we present a comprehensive review of HCMV-specific mAbs isolated from the 1980s to the present, detailing their target antigens, epitopes, neutralizing activity, and potential clinical applications. This review integrates historical discoveries with recent structural and functional studies, highlighting trends that may inform the development of anti-HCMV therapeutics and prophylactics.

## INTRODUCTION

### Human cytomegalovirus (HCMV)

HCMV is an ancient human β-herpesvirus that is widespread worldwide (1). Having coevolved with humans for over 100 million years, it offers a unique opportunity to study immune defense strategies and pathogen counterstrategies. HCMV triggers both humoral and cell-mediated immune responses (1, 2). Despite a robust anti-HCMV immune response, the virus establishes persistent infections in more than 90% of the population, with latency in hematopoietic progenitor cells (1–3). While HCMV infections are typically asymptomatic in immunocompetent populations, they pose a significant risk to immunocompromised individuals (4). Particularly, neonates, whose immature immune systems predispose them to infections (4–7), solid organ and stem cell transplant recipients (8, 9), and those with acquired immunodeficiency syndrome (AIDS) (10–12), where the morbidity associated with this virus can be high. HCMV disease in transplant recipients is most characterized by fever, malaise, leukopenia, thrombocytopenia, and upper digestive tract pain. Progression to multi-organ dysfunction can result in mortality (13). Unfortunately, available prophylactics and therapeutics do not prevent and treat HCMV infections in all affected populations.

### Current therapeutics and prophylactics targeting HCMV

Small-molecule antivirals have had some success in managing HCMV viremia in transplant recipients. The current standard of care includes ganciclovir and its prodrug, valganciclovir, both of which function as nucleoside analog inhibitors (14). However, both molecules have been shown to cause neutropenia, thereby further weakening the immune systems of already immunocompromised hosts. Additionally, both molecules are potential teratogens and, therefore, not appropriate for use to prevent congenital HCMV (cCMV) transmission. While the majority of HCMV disease in transplant recipients now occurs after discontinuation of ganciclovir, mutations leading to resistance to ganciclovir have been well characterized and increase the risk of poor outcomes in transplant recipients (15). Escape mutations have also proven to be a problem for the new antivirals letermovir (16) and maribavir (17), which were approved for the prevention of HCMV infection and disease in adult transplant recipients in 2017 (18) and 2021 (19), respectively. Maribavir was additionally approved for children over 12, making it one of the few anti-HCMV antivirals that can be used in pediatric transplant recipients (17, 19).

Treatment of cCMV has been limited to passive hyperimmune globulin immunization (20), marketed as Cytotect in Europe and Cytogam in the United States. While this is generally well tolerated by the patients, with adverse effects reported in <6.0% of cases (21), its cost can be prohibitive for populations of low socioeconomic status. Additionally, studies in 2014 (22) and 2018 (23) have called into question the efficacy of this treatment option. This is further complicated by Cytogam-dosing regimens being based on recipient weight, rather than on standardized CMV IgG content or measured activity (21), making it difficult to quantify the benefits or detrimental effects of such exogenous antibodies.

### Neutralizing antibodies

Neutralizing antibodies (nAbs) targeting viral glycoproteins are defined as Abs that inhibit viral entry by directly binding viral antigens (24–27). In some cases, this binding—directly or sterically—prevents the virus from interacting with the appropriate host-cell receptor (28–30); however, in other cases, these nAbs have been known to prevent conformational rearrangements necessary for fusion (31, 32). Neutralization is mediated by the fragment antigen-binding (Fab) portion of an Ab. It is generally classified as an *in vitro* property, as it can be tested only in a minimal system to ensure that all phenotypes result directly from virus-Ab interactions. nAbs have been successfully developed for safe and effective passive immunization against viral infections and diseases, such as respiratory syncytial virus (RSV), Ebola, and Severe Acute Respiratory Syndrome Coronavirus 2 (SARS-CoV-2) (24, 31, 33). Neutralization alone does not always reliably predict *in vivo* efficacy, however. Indeed, for HCMV, neutralization has not yet been shown to correlate with protection against acquisition, vertical transmission, or disease.

### Antibody effector functions

In contrast to neutralization, which is mediated by the Fab portion of an Ab, the non-neutralizing, or effector, functions of Abs are mediated by the crystallizable fragment (Fc) portion of the Ab. This is the constant, “stem-like” portion of an Ab that does not directly bind to antigens but, instead, interacts with certain host cell receptors. Antibody effector functions include antibody-dependent cellular cytotoxicity (ADCC), antibody-dependent cellular phagocytosis (ADCP), and complement-dependent neutralization (CDN), among others. A detailed overview of these functions, the cell types involved, and their role in viral immunity can be found in the 2023 review by Chandler et al. (34). In viruses such as Ebola, HSV, and influenza A, effector functions have been shown to correlate with enhanced protection against disease (35–37).

In HCMV, the correlates of protection have yet to be fully characterized. However, effector functions such as ADCC and complement-dependent responses, such as complement-dependent neutralization and complement-enhanced neutralization, have been associated with better clinical outcomes in congenital HCMV infections (38–43).

### Overview

Some of the earliest studies in the field of HCMV sought to characterize antibody responses to this virus, leading to the generation of large panels of murine monoclonal antibodies (mAbs) that continue to be used as gold-standard controls. These early efforts laid the foundation for our current understanding of the HCMV antigenic structure. In recent decades, technological advances in B-cell isolation, recombinant expression, and structural biology have enabled the discovery and characterization of highly potent human mAbs directly from infected individuals and vaccine recipients. This review compiles these findings in a structured format, organized by target antigen, and examines how both historical and more recent mAbs can inform ongoing therapeutic and vaccine efforts.

**Fig 1:**
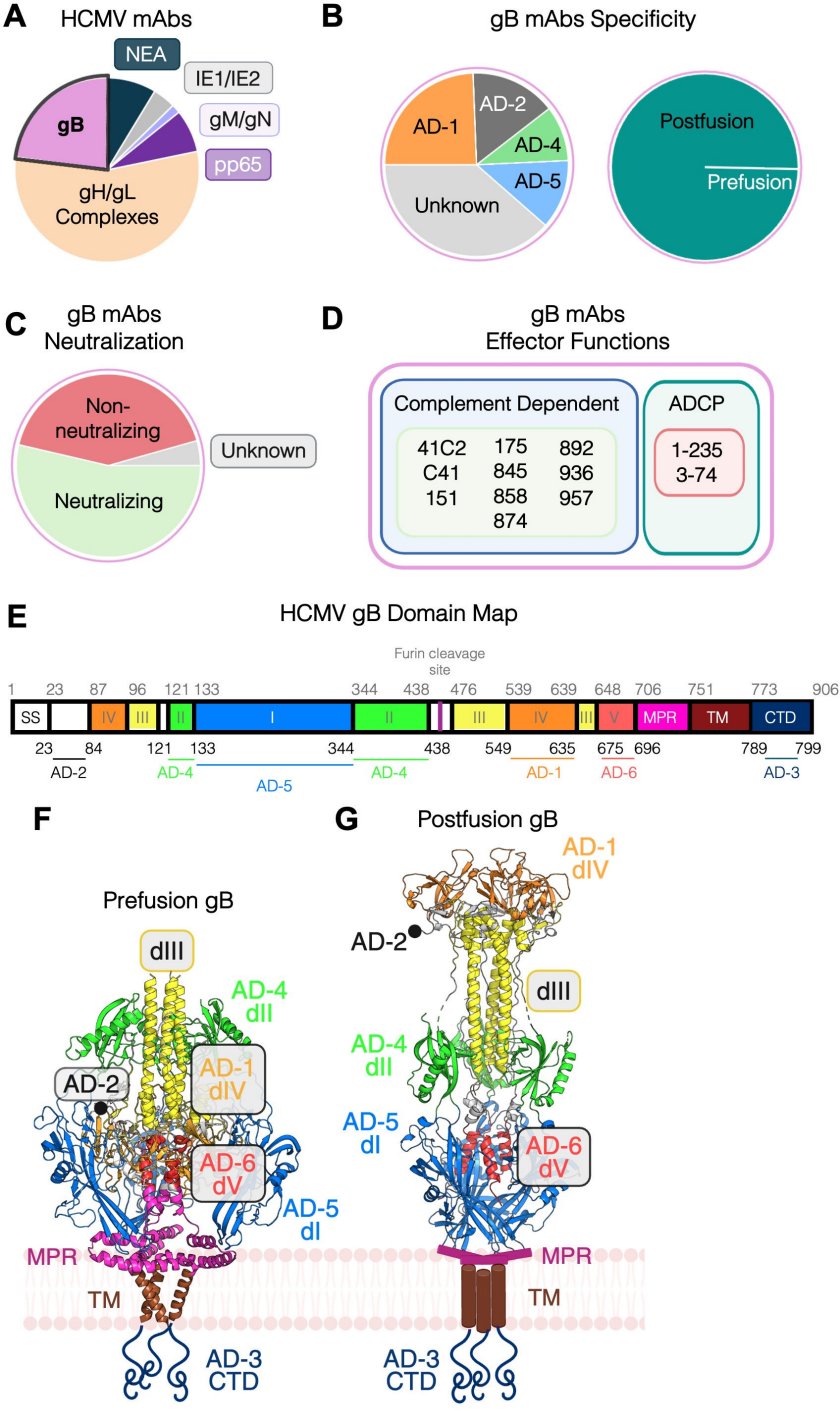
Nearly a quarter of all isolated HCMV mAbs target gB. (**A**) Pie chart representing the percent of all HCMV mAbs that have been isolated; 22.8% of these (205/901) target gB. Non-envelope antigens abbreviated as “NEA.” (**B**) (Left) All gB mAbs are grouped by their target antigenic domain, in accordance with colors in (E–G): antigenic domain 1 (AD-1) (50/205, 24.4%); AD-2 (31/205, 15.1%); AD-4 (20/205, 9.8%); AD-5 (25/205, 12.2%); and mAbs with unknown domain specificity (79/205, 38.5%). No mAbs have been published against AD-3 or AD-6. (Right) All gB mAbs are grouped by their target conformation: postfusion (204/205, 99.5%) and prefusion (1/205, 0.5%). (**C**) All gB mAbs are grouped by their neutralization properties: neutralizing (110/205, 53.6%); non-neutralizing (86/205, 42.0%); and mAbs with unknown neutralization properties (9/205, 4.4%). (**D**) gB mAbs with known effector functions: complement-dependent neutralization or ADCP. Neutralization properties are colored in accordance with panel C. (**E**) A schematic view of HCMV gB structural and antigenic domains. Structural domains (dI-dV) of the ectomain, membrane-proximal (MPR), transmembrane (TM), and cytoplasmic (CTD) domains are colored in accordance with panels F and G. Antigenic domains (AD-1–AD-6) are indicated underneath the diagram. (**F**) Structure of prefusion gB (RCSB: 7KDP) and (**G**) structure of postfusion gB (RCSB: 5CXF). Structural domains and antigenic domains within are colored and labeled. The terminal residue of AD-2 is represented by a black circle on one monomer of gB. AD-3 is represented by navy blue lines. Membrane proximal region (MPR) of postfusion gB is represented by magenta cylinders. Transmembrane region (TM) of postfusion gB is represented by brown cylinders. For detailed information on individual mAb clones, see Table S1.

Although HCMV has nearly twenty glycoproteins in its envelope (44), known mAbs target only a small subset of these. We will begin with glycoprotein B (gB), the viral fusogen that is conserved across all herpesviruses, which was the first HCMV antigen to be studied and remains a major target of anti-HCMV mAb response (Fig. 1A). Next, we will introduce the gH/gL heterodimer and its HCMV-specific complexes, trimer (gH/gL/gO), pentamer (gH/gL/UL128/130/131A), and the newly identified GATE complex (gH/UL116/UL141), which bind host-cell receptors, determine HCMV tropism, and elicit most of the anti-HCMV mAbs (Fig. 1A). Finally, we will present the most abundant glycoprotein complex on the surface of HCMV, gM/gN, for which few antibodies have been isolated (Fig. 1A). A significant subset of anti-HCMV antibodies has been isolated against non-enveloped proteins, such as Immediate Early 1 (IE1), IE2, and tegument proteins, which round out this review. We hope that this comprehensive overview of HCMV-specific monoclonal antibodies, their associated antigens, and the references herein will help advance the field.

## GLYCOPROTEIN B

### Glycoprotein B structure and function

Glycoprotein B (gB) is a central target of the adaptive immune response, with ~200 mAbs reported (Fig. 1A and B; Table S1), many of which demonstrate functional properties like neutralization and effector functions (Fig. 1C and D; Table S1). gB functions as a fusogen. In enveloped viruses, fusogens are essential viral surface glycoproteins that mediate the fusion of the viral envelope with the host membrane during entry and cell-to-cell spread. They achieve this biophysical feat by refolding from the metastable prefusion to the stable postfusion form. Energy released during this refolding process is thought to overcome the large kinetic barrier to fusion (45). gB homologs are found in all *Orthoherpesviridae* and are class III fusogens, sharing this structural classification with the glycoprotein (G) of vesicular stomatitis virus (VSV) and gp64 of baculoviruses (46). Potentially because of the metastability of the prefusion form of gB, only one anti-gB mAb isolated to date preferentially binds the prefusion form (Fig. 1B). When co-expressed with gH/gL, gB is necessary and sufficient for epithelial cell-cell fusion (47). Additionally, a hyperfusogenic chimera of gB can mediate cell-cell fusion alone (48), highlighting the importance of this protein in facilitating membrane fusion during target cell entry.

Historically referred to as gC-I (49), gp58 (50, 51), or gp55-116 (52–55), HCMV gB is encoded by the *UL55* gene. HCMV gB is a ~900 amino acid protein composed of a large ectodomain, a membrane-proximal region, a transmembrane helix, and a cytoplasmic domain (56, 57) (Fig. 1E through G). The ectodomain contains a furin cleavage site that cleaves gB into two polypeptides, a 116 kDa N-terminal fragment and a 55–58 kDa C-terminal fragment, which were the basis of its historical nomenclature (58, 59) (Fig. 1E). The gB ectodomain consists of five structural domains (dI–dV) that undergo large rearrangements during pre- to post-fusion transition, while preserving their overall folds (Fig. 1F and G). In both pre- and post-fusion forms, gB is a homotrimer.

Thus far, six antigenic regions, referred to as antigenic domains (ADs), have been defined. Four of these, AD-1, AD-4, AD-5, and AD-6, are each located within a corresponding structural domain (Fig. 1F and G). The ADs are described in more detail below.

### Antigenic domain 1 (AD-1)

AD-1, located within the structural domain IV (dIV) (Fig. 1F and G), was the first AD to be defined because some of the first anti-gB nAbs targeted this region (60). It is a continuous polypeptide in the C-terminal fragment of dIV, spanning residues 549–635 (Fig. 1E). Given its continuous nature, it is not surprising that a significant majority of AD-1 mAbs possess linear epitopes, and their discovery was based on western blot analysis (Table S1). Many of the AD-1 mAbs are potent neutralizers, although most have been tested only against the AD169 strain of HCMV due to their early discovery. Of note are AD-1-targeting mAbs 7–17 (61) and mAbs from the ITC series (62). In 1984, the first set of murine anti-gB mAbs was isolated by Britt (61). This panel included mAb 7–17, which is neutralizing and binds to a linear epitope on AD-1. Since then, this nAb has been used extensively in HCMV gB studies. mAbs from the ITC series isolated by Ohlin et al. represent the first large panel of human-derived mAbs against HCMV gB. This set includes neutralizing and non-neutralizing mAbs, the majority of which bind AD-1, with a single mAb binding gH/gL (62).

AD-1 contains the largest number of published complement-dependent nAbs (Fig. 1D; Table S1), largely due to a 2021 study (63) that isolated mAbs from six subjects vaccinated with V160, an experimental HCMV vaccine candidate derived from AD169 strain with the pentamer restored (64). The majority of anti-gB mAbs elicited in this study were against AD-1, and most were non-neutralizing. However, those that were neutralizing were complement-dependent, sometimes exhibiting >100-fold increase in neutralization in the presence of rabbit complement. Although V160 ultimately did not meet the Phase 2 efficacy endpoint standards (65), these mAbs could be used as controls in future studies.

The clinical relevance of AD-1 was revisited in 2023 with the publication of a highly neutralizing mAb, EV2038 (66). This nAb neutralized four laboratory strains (AD169, Towne, Davis), a low-passage clinical isolate (Merlin), and 42 clinical isolates of Japanese origin, with IC_50_s ranging from 0.013 to 0.105 μg/mL, which is on par with anti-pentamer nAbs that are considered the most potent in the field and are discussed in the corresponding section of this review. Okamoto et al. also demonstrated the potency of EV2038 in reducing cell-to-cell spread of eight clinical isolates. The conformational epitope of EV2038 spans three discontinuous sequences—residues 549–560, 569–576, and 625–632. These three sequences were highly conserved across 71 clinical strains, providing the basis of the broad neutralization ability of EV2038 and further characterization of the most significant antigenic sub-region of AD-1. In a study rare for the HCMV Ab characterization, Okamoto et al. tested the pharmacokinetics of EV2038 in Cynomolgus macaques and showed that its serum levels remained high for up to 28 days after administration. As this is a recently published mAb, no clinical trials have yet been conducted. Nonetheless, this study shows promise for AD-1-specific nAbs as therapeutic candidates.

### Antigenic domain 2 (AD-2)

The first AD-2 mAb, C23, was isolated in 1987 (67) and later shown to be a complement-independent nAb (68). However, AD-2 was not defined until 1990, when Meyer et al. used this mAb, C23, to map this antigenic region (68). AD-2 encompasses N-terminal amino acids 23 to 84(68, 69) (Fig. 1E). A study on congenital HCMV (cCMV) transmission initially reported results indicating a weak correlation between an IgG response to AD-2 and reduced cCMV transmission (70). However, a follow-up plasma anti-gB-AD-2 IgG blocking assay in the same study revealed no such association (70). An additional study hypothesized that mAbs against AD-2 can only develop through rare rearrangement events (71), highlighting its unique characteristics. AD-2 is also the target of 1–235, one of two anti-gB mAbs that have demonstrated ADCP activity *in vitro* (72, 73) (Fig. 1D; Table S1).

AD-2 contains two antigenically dominant sites: the first, Site I, is located between amino acids 68 and 77 (69). AD-2 Site I is conserved between the laboratory strains AD169 and Towne, as well as low-passage clinical isolates (69). AD-2 is targeted by mAb C23 (69). In the 1990s, C23 was rebranded as TI-23 (Regavirumab) and tested as a potential therapeutic in Japan (74). However, these trials were stopped in 1999, and C23 is now commercially available as a laboratory reagent.

In 2014, TRL345, a highly neutralizing mAb against AD-2 Site I, with an EC_50_ of 0.07–0.42 μg/mL, depending on cell type, was reported by Trellis Bioscience (75). TRL345 has a picomolar affinity for AD-2 Site I, which is 10-fold higher than that of a previous AD-2 Site I nAb, TCN-202, which failed a Phase 1 clinical trial (76). TRL345 neutralized infection in fibroblasts, smooth muscle cells, epithelial, and endothelial cells against 15 clinical strains of HCMV (75). Additionally, TRL345 prevented cell-to-cell spread in epithelial cells and HCMV infection in placental explants (75). Most curious, perhaps, is that the frequency of mutations at the epitope of TRL345 is ~10-fold lower than at other envelope glycoprotein sites (77, 78), suggesting an evolutionary need to conserve this epitope. This mAb is currently being prepared for use in human patients, and a comprehensive review of its properties was published in 2018 (78).

The frequency of naturally occurring mAbs against AD-2 Site I has been debated. In 1992, a study examining HCMV-positive human sera reported that >50% of samples recognized AD-2 Site I (69). Subsequent investigations citing this original work reported AD-2 as an immunogenically dominant region (70, 79). However, in 1997, two studies reported that only a very small subset of seropositive individuals develops such mAbs, often with delayed kinetics (80) and low titers (81). More recent studies also reported AD-2 Site I to be poorly immunogenic (78). Nonetheless, it is notable that mAbs targeting AD-2 Site I have been associated with better disease outcomes after solid organ transplantation (79).

Unlike AD-2 Site I, Site II is minimally conserved across strains and is located between residues 50 and 54. mAbs targeting this region are not neutralizing *in vitro*. One of these mAbs, CH408, exhibited some neutralization in the presence of complement, but the extent of neutralization was not considered physiologically relevant (69). It is important to note that although all AD-2 Site II mAbs were tested in the presence of complement, they were tested with 0.5% complement, a concentration 10-fold to 25-fold lower than that used in other studies, and only against AD169 (61, 69, 82). Revisiting these clones at higher complement concentrations may yet uncover their complement-mediated properties.

### Antigenic domain 3 (AD-3)

AD-3 is located within the intraviral/cytoplasmic domain of gB, spanning residues 798–805 (83) (Fig. 1E through G). This region was discovered when sera from HCMV-seropositive blood samples were tested for reactivity against synthetic peptides, and a significant majority reacted to the 798–805 peptide, which defined AD-3. To our knowledge, no AD-3-specific mAbs have yet been isolated (Fig. 1B), however. This is likely due to their inability to neutralize the infection, given the intraviral location of the AD-3. As a result, AD-3-directed antibodies are abundant but expected to be non-neutralizing, consistent with recognition of a region of gB inaccessible on intact virions. Its immunodominance in naturally infected individuals likely arises from intracellular processing, protein turnover, and cell lysis, which expose this otherwise cytoplasmic, linear epitope to the immune system. Studies on AD-3 remain limited to its original discovery (83) and a 2019 study examining serum reactivity to all previously discovered ADs (84, 85).

The 2019 study by Baraniak et al. examined the sera from individuals previously vaccinated with gB/MF59 and found that 76% of available samples were reactive to AD-3 (85). AD-3 could thus be an example of an accidental antigenic decoy. In natural infection, antigenic decoys are an immune-evasion strategy in which viruses shed or secrete glycoproteins or their fragments to divert attention from the primary form. These secondary secreted forms act as antigenic “sinks” that absorb Abs and divert responses from functional targets (86, 87). The cytoplasmic location of AD-3 would prevent it from serving as a true decoy. However, AD-3 was immunologically exposed in the gB/MF59 vaccine due to the use of a soluble gB construct containing both the ecto- and the intra-viral/cytoplasmic domains. gB/MF59 is discussed in further detail later. The unexpectedly strong immunodominant response against AD-3 may have diverted humoral responses away from protective epitopes on gB (84). The authors of this study proposed that future vaccine candidates exclude AD-3 to elicit a greater magnitude and breadth of nAb responses.

### Antigenic domain 4 (AD-4)

Antigenic domains 4 and 5 were identified in the same 2011 study by Pötzsch et al. and have since been established as antigenically dominant regions of gB, capable of eliciting robust nAbs (88–91) (Fig. 1B). AD-4 is located within the structural domain II (dII) and is discontinuous, being composed of residues 121–132 and 344–438 (Fig. 1E). AD-4 mAbs were found in >90% of seropositive donor samples used in the 2011 study (88) and capable of neutralizing infection of fibroblasts, endothelial, epithelial, and dendritic cells (88). The most robust AD-4 nAb, SM5-1, neutralized all four cell types with IC_50_s ranging from 0.2 to 0.3 μg/mL. This nAb has been used extensively in subsequent studies to characterize AD-4 and as a robust control for neutralization studies. Further studies of AD-4 in 2013 (92) revealed a smaller epitope within the antigenic region required for many AD-4 mAbs to bind properly. Substitution of Tyr364 and Lys379, termed the “YK epitope,” resulted in partial or complete disruption of the binding of mAbs isolated within the 2011 study. Recombinant viruses containing gB with a mutated YK epitope were severely replication-deficient in fibroblasts (89). Therefore, the YK epitope is likely both functionally and antigenically important for HCMV (89). Given the immunological significance of this antigenic region, it is perhaps not surprising that one of the earliest anti-HCMV cocktails, CSJ148 (93), contained an AD-4 nAb, LJ538, originally named 7H3(94). This cocktail is discussed in further detail in the gH/gL section of this review. The structures of 7H3 bound to postfusion and prefusion-like gB, gB-C7, were solved in 2024 (95).

### Antigenic domain 5 (AD-5)

AD-5 was initially discovered in 2011 (88), but its most extensive characterization thus far was conducted in the 2025 study by Wu et al. (90). It is composed of residues within the structural domain I (dI) of HCMV gB, residues 133–343 (88, 90, 96) (Fig. 1E through G). Unlike AD-4 mAbs, AD-5 mAbs isolated in 2011 potently neutralized infection of fibroblasts, endothelial, and epithelial but not dendritic cells. The most potent nAb from the 2011 study, 1G2, is now considered a canonical AD-5 mAb and has been used extensively.

Wu et al. also isolated 16 new, highly potent AD-5 nAbs (Table S1) and showed that mice immunized with a recombinant AD-5 produced higher bulk titers of nAbs than mice immunized with full-length gB (90). This finding suggests that future HCMV vaccine designs should explore the use of recombinant antigenic regions rather than whole proteins.

### Antigenic domain 6 (AD-6)

AD-6 is located within the structural domain V (dV), spanning residues 675–695 (97) (Fig. 1E through G). In this work, the authors showed that seronegative participants in the gB/MF59 vaccine trial who later received solid-organ transplants exhibited reduced viremia if they had developed Abs against AD-6 prior to transplantation. Abs against AD-6 were rarely detected in naturally infected individuals but were enriched in post-gB/MF59 vaccine samples. Given the location of AD-6, this may be due to the transmembrane region being removed from the gB/MF59 antigen (98), thereby allowing greater exposure of a typically buried region (Fig. 1F and G). To date, no mAbs have been isolated against this region (Fig. 1B). Curiously, polyclonal antibodies (pAbs) against AD-6 did not neutralize HCMV in fibroblasts or epithelial cells, with or without complement. However, these pAbs dramatically reduced the cell-to-cell spread of HCMV strain Merlin.

Abs against AD-6 were rarely detected in naturally infected individuals but were enriched in post-gB/MF59 vaccine samples. Given the location of AD-6, this may be due to the transmembrane region being removed from the gB/MF59 antigen (98), thereby allowing greater exposure of a typically buried region (Fig. 1F and G). To date, no mAbs have been isolated against this region (Fig. 1B). Nonetheless, AD-6 shows promise as a protective antigenic target. It also serves as a persuasive example of revisiting and reanalyzing older samples and clones to advance the field, as well as of isolating new candidates.

### gB/MF59 vaccine candidate

The first recombinant HCMV vaccine, gB/MF59, was developed in the 1990s. It consisted of a near-full-length gB from HCMV strain Towne, containing the ectodomain and intraviral/cytoplasmic domains but lacking the transmembrane region, which was removed to facilitate efficient secretion (98, 99). We refer to this soluble gB variant henceforth as gBΔTM. It also contained the MF59 adjuvant. Although the vaccine induced high titers of nAbs during the Phase 1 clinical trial (98) in healthy participants, it was discontinued after Phase 2 because maximum efficacy was only 50% in transplant patients, with lower nAb titers likely due to the immunosuppressive anti-rejection drugs (100). All gB/MF59 clinical trial cohorts excluded pregnant women, anticipating that this high-risk population would be included in Phase 3. A comparison of gB/MF59 and other HCMV vaccine candidates is outside the scope of this review but can be found in this comprehensive review (101).

Of interest, however, is how gB/MF59 has aided in the identification and characterization of antigenic regions of gB. Following the discontinuation of this vaccine candidate, sera from vaccinated seronegative individuals from Phase 2 who received organ transplants after vaccination were reanalyzed to determine which serum properties correlated with protection. NAb responses did not correlate with protection because responses to the five previously identified antigenic regions were minimal (85, 102). The authors then investigated ADCC activation because it had been shown to be correlated with protection against influenza but had not yet been implicated in HCMV transmission (102, 103). However, no evidence of ADCC activation was found in the post-vaccination serum samples from solid organ transplant recipients (102).

It is unclear what underlying mechanism accounted for the 50% efficacy observed during Phase 2. Some light was shed in 2020 in a study that utilized Phase 2 serum samples to investigate the correlates of protection in postpartum and adolescent women (104). Protection against primary HCMV infection correlated with serum IgG binding to cell-associated gB, but not to the soluble vaccine antigen gBΔTM. This likely reflects structural differences between the two forms: soluble gBΔTM exposes immunodominant epitopes that are normally inaccessible in the native, membrane-associated protein, such as AD-3, which may act as antigenic sinks, diverting the immune response toward non-functional targets. In contrast, antibodies elicited against cell-associated gB are more likely to recognize epitopes more relevant to the fusogenic activity of gB, thereby contributing to functional neutralization. Although this study clarified why functional nAb responses were lower than expected with gB/MF59 vaccination, it remains unclear what accounted for the 50% efficacy observed in Phase 2.

Additional investigations using gB/MF59 Phase 2 samples also led to the discovery of the newest antigenic region, termed Antigenic Domain 6, in 2023 (97), as described above.

## gH/gL AND ITS COMPLEXES

**Fig 2:**
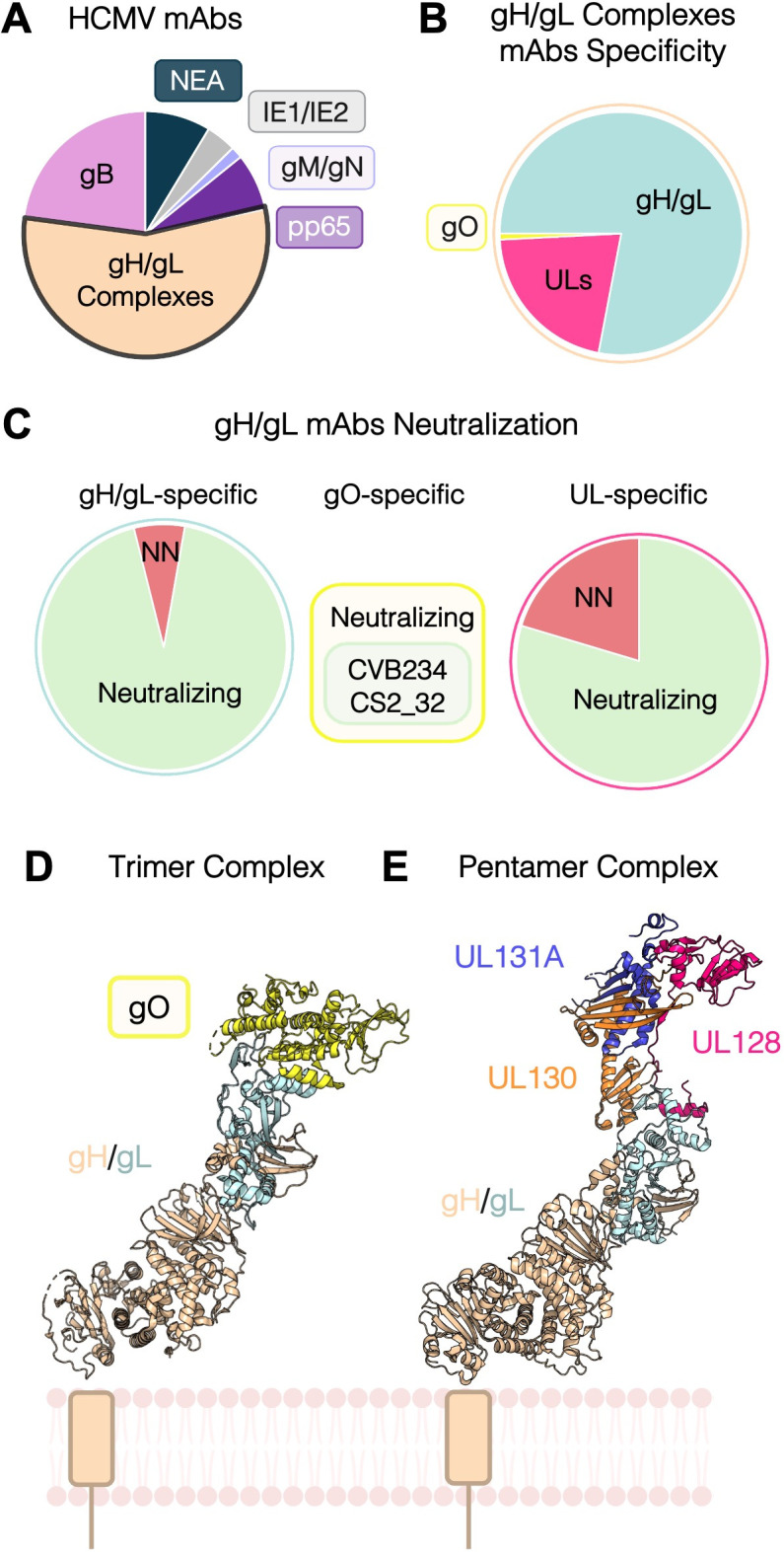
Most isolated anti-HCMV mAbs target gH/gL complexes. (**A**) Pie chart representing the percent of all HCMV mAbs that have been isolated; 55.5% of these (500/901) target gH/gL complexes. Non-envelope antigens abbreviated as “NEA.” (**B**) All mAbs targeting gH/gL complexes are grouped by their specific antigenic target: gH/gL-specific mAbs (390/500, 78.0%); mAbs specific to UL128/UL130/UL131A (“ULs,” 106/500, 21.2%); and gO-specific mAbs (4/500, 0.8%). (**C**) All mAbs targeting gH/gL complexes, grouped by their specific antigenic target, are further grouped by their neutralization properties. (Left) gH-gL-specific mAbs: neutralizing (364/390, 93.3%) and non-neutralizing (NN) (26/390, 6.7%). (Middle) Only two gO-specific mAbs are neutralizing (2/4). (Right) UL-specific mAbs: neutralizing (83/106, 78.3%) and NN (23/106, 21.7%). (**D**) Structure of the trimer complex (RCSB: 8TCO). Transmembrane and cytoplasmic tails of gH are represented by wheat rectangles and lines. (**E**) Structure of the pentamer complex (RCSB: 5VOB). Transmembrane and cytoplasmic domains of gH are represented by wheat rectangles and lines. For detailed information on individual mAb clones, see Table S2 (gH/gL), S3 (gO), and S4 (ULs).

In all *Orthoherpesviridae*, gH/gL is required for entry into the target host cells along with gB, due to its role in fusion regulation. In herpes simplex viruses 1 and 2, gH/gL binds the receptor-binding protein, gD, after gD has bound the target cell receptor, such as nectin-1 (105–107). gH/gL then somehow activates the fusogenic ability of gB, potentially through interactions between the cytoplasmic domains of gH and gB (108). In γ-herpesviruses Epstein-Barr Virus (EBV) and Kaposi’s Sarcoma Herpesvirus (KSHV), gH/gL either binds a receptor-binding protein, such as EBV gp42, or directly engages the host cell receptor (105, 109). In β-herpesviruses, gH/gL forms two distinct complexes: the pentamer, which contains UL128, UL130, and UL131A; and the trimer, which contains gO (105, 110–112). These complexes facilitate HCMV entry into distinct cell types, as discussed in the sections corresponding to each complex. A significant subset of mAbs isolated using the pentamer or trimer has been found to interact with both complexes due to their specificity for gH (Fig. 2A and B; Table S2), and a majority of these mAbs demonstrate functional properties (Fig. 2C, left; Table S2). These Abs are distinct from the pentamer- or trimer-specific Abs and will be discussed separately.

### gH/gL structure and function

HCMV glycoprotein H (gH), historically referred to as gp86 (113, 114), is a ~90 kDa type I transmembrane protein encoded by the UL75 gene (105, 106, 111). gH is typically covalently bound to the soluble ~25 kDa glycoprotein L (gL), encoded by the UL115 gene (115) (Fig. 2D and E). Residue C95 of gH forms a disulfide bond with residue C47 of gL (116). However, in the newly identified GATE glycoprotein complex, gH is, instead, bound to UL116, which is, in turn, is bound to UL141 (117, 118). The binding of gH to gL or UL116 is required for correct folding, trafficking to the cell surface, and the function of both proteins. In the absence of gL or UL116, gH fails to mature beyond the ER (115, 117). When overexpressed in uninfected cells, gH/gL and gB can promote cell-cell fusion of ARPE-19 retinal epithelial cells (47). Efficient cell-cell fusion, however, also requires gO (119, 120).

Zehner et al. proposed that gH/gL be subdivided into six antigenic domains (ADs) (29). Similar to the ADs of gB described above, these were identified by clustering mAbs targeting epitopes within gH/gL. For gB, the relative immunogenicity of its ADs has been partially defined, but the same cannot yet be said for gH/gL. Continued use of AD-nomenclature for gH/gL may help classify both newly isolated and historical mAbs more accurately.

### Overview of gH/gL antibodies

To date, 500 mAbs targeting the gH/gL or gH/gL-containing complexes have been published, with the majority binding to gH (Fig. 2A and B; Tables S2 to S4). The first of these mAbs, 1G6, reported in 1984, is a neutralizing murine mAb specific for gH (82). Although 1G6 was the first, another mAb isolated in 1989, 14-4b (121), remains the most commonly used control mAb for studies of HCMV gH/gL. The study that reported 1G6 is notable, however, because it tested the effect of complement on the neutralization ability of 1G6. Since then, none of the other anti-HCMV gH/gL mAbs have been tested for effector functions, seemingly due to a shift within the field towards viewing neutralization as a better predictor of protection. Indeed, nearly all recent HCMV mAb studies have characterized neutralization properties of newly discovered mAbs but not their effector functions.

### MSL-109: a case study

One of the best-known anti-gH mAbs, MSL-109 (122), provides an excellent case study of the potential pitfalls associated with the use of nAbs as therapeutics and reliance on neutralization as a predictor for *in vivo* outcomes. MSL-109 is a human IgG1 that was isolated in the early 1990s from a seropositive individual (122, 123). It neutralizes multiple strains of HCMV, including the lab-adapted strains AD169 (124) and Towne (125) and clinical isolate strains (123, 125). Given its broad tropism, this mAb was chosen for a clinical study targeting HCMV retinitis (11). HCMV-associated retinitis affects nearly 20%–40% of individuals living with acquired immune deficiency syndrome (AIDS) (10). It is characterized by initially mild symptoms that can progress to irreversible vision loss due to retinal detachment (12). Despite promising *in vitro* data, this mAb proved ineffective in patients, and development was halted before Phase 3 of the clinical trial (11).

Shortly thereafter, a study suggested that the virus could have developed resistance to MSL-109 (126), but no mechanism was proposed until 2011 when Manley et al. elucidated a nongenetic escape mechanism (127). This landmark study showed that mAbs could be selectively incorporated into assembling virions, thereby yielding virions coated with MSL-109 mAbs. The virus then used the Fc portions of virion-bound mAbs to enter nearby cells via clathrin-dependent endocytosis, a process that required Fc domain availability but did not appear to involve classical Fcγ receptor-mediated uptake, suggesting the engagement of a noncanonical Fc-dependent entry pathway (127). Notably, MSL-109 promotes entry rather than blocking it, as it binds a cell-associated gH epitope and is incorporated into assembling virions, thereby repurposing its Fc domain to facilitate viral uptake rather than prevent fusion (127).

To improve neutralization potency, MSL-109 was affinity-matured into MCMV5322A, thereby increasing its affinity for gH by more than 10-fold (128, 129). However, as the MSL-109 resistance mechanism is driven by Fc-dependent viral entry following antibody incorporation into virions, increased Fab affinity alone would not be expected to prevent this mode of escape. To mitigate this limitation, MCMV5322A was combined with a pentamer-specific mAb, MCMV3068A, to form the cocktail RG7667 (129). This combinatorial approach was thought to increase the barrier to resistance by targeting distinct entry pathways, thereby reducing the likelihood that Fc-mediated escape of a single antibody could sustain productive infection. Although the resistance mechanism of MSL-109 was unprecedented in HCMV, it has been observed before in Dengue virus infection, where it is termed “antibody-dependent enhancement” (ADE). Secondary infection with a heterologous serotype of Dengue significantly increases the risk of severe disease as a result of ADE (130). MSL-109 serves as a cautionary tale against relying solely on *in vitro* neutralization ability as the sole predictor of *in vivo* outcomes.

### Contemporary anti-gH/gL-neutralizing antibodies

Following the series of anti-gH/gL mAb isolations in the 1990s, it was not until 2010 that Macagno et al. isolated the first group of anti-gH/gL mAbs from human donors (94) (Table S2). In addition to providing the field with mAbs such as anti-gH/gL 13H11, frequently used as a reagent in functional and structural studies, this study was one of the first to test neutralization in both fibroblasts and epithelial cells. Previously, mAbs were tested for neutralization only in fibroblasts. However, the nAbs were far more potent in epithelial cells, with IC_90_s up to 5-fold lower than in fibroblasts. Additionally, this study reintroduced gH/gL, particularly the pentamer complex, as an antigen worth investigating, thereby sparking a new wave of mAb isolations.

Between 2021 and 2023, a remarkable 343 mAbs were isolated and published against gH/gL (Table S2), with the majority of these coming from just two studies (29, 63). Collectively, there are now nearly 400 anti-gH/gL mAbs (Fig. 2B), most of them human and neutralizing (Fig. 2C, middle; Table S2). However, neither the effector functions nor the host cell interactions of these mAbs have yet been characterized. Heeding the cautionary tale of MSL-109, the first gH/gL mAb, it would be worth characterizing the effector functions and host-cell interactions of all these mAbs. A recent study on the Ebola virus systematically characterized existing mAbs against its surface glycoprotein (GP), thereby identifying features that contribute to protection (35). This study found that effector functions, particularly ADCC, were among the correlates of protection. The identification of correlates of protection has been a longstanding gap in the field of HCMV; hence, performing a comparable analysis of the hundreds of anti-HCMV mAbs would provide valuable information.

### Trimer structure and function

The trimer glycoprotein complex, historically referred to as gC-III (131, 132), consists of gH/gL and another glycoprotein, gO (Fig. 2D). gO, originally referred to as gp125 (131), is a soluble glycoprotein encoded by the UL74 gene (132), with approximately 18 N-linked glycosylation sites (111). Due to the abundance of glycans on its surface, it migrates at approximately 125 kDa, reflected in its historical name. However, its unmodified form is predicted to be about half that size (131, 132). gO forms a disulfide bond with residue C144 of gL (116). The high-resolution structures of the gH/gL/gO trimer were reported in 2021 (133, 134) (Fig. 2D).

Trimer facilitates HCMV entry into fibroblasts, epithelial cells, and endothelial cells through interactions between gO and PDGFRα (135). Although it was initially thought to modulate entry into fibroblasts exclusively, that view has since been overturned. Instead, the trimer is now accepted to be important for entry into all cell types (136). The surface expression of trimer varies significantly from strain to strain, with strains with higher expression levels showing proportionally increased entry efficiencies (136, 137). This relationship was defined using replication-deficient adenovirus (Ad) vectors, in which strain-specific differences in gO surface expression were directly measured (137). Mutant viruses lacking the trimer produced extracellular viral particles yet failed to infect fibroblasts, epithelial, and endothelial cells (138). While several laboratory-passaged strains, including AD169, have been reported to contain defects in the other gH/gL complex, the pentamer (112, 139), a few have been reported to contain entry-diminishing mutations in the trimer complex (137), highlighting the importance of this complex to HCMV function.

### Overview of the anti-trimer antibodies

Despite its significance to HCMV biology, most studies attempting to isolate mAbs against gH/gL/gO have only succeeded in obtaining anti-gH/gL mAbs. This could be, in part, due to the heavy glycosylation of gO. To date, only four mAbs specific to gO have been reported (Fig. 2B; Table S3), two of which are neutralizing (Fig. 2C, middle; Table S3). In 2016, Gerna et al. isolated human mAbs CVB234 and CVB301, the former of which was the first anti-gO nAb (30). CVB234 neutralized HCMV with an IC_50_ of 1–3 μg/mL (30), making it about 10-fold less potent than the most recently published CS2it1p2_F7K (aka CS2_32), which has an IC_50_ of 133.33 ng/mL (29) (Table S3). CS2it1p2_F7K is also the only anti-gO mAb for which a high-resolution cryo-EM structure, in complex with the trimer, is known (29). Despite their modest numbers, the properties of these mAbs make them valuable reagents for downstream investigations into the trimer complex.

### Pentamer structure and function

Pentamer, or the pentamer complex (PC), contains gH/gL bound to UL128/UL130/UL131A (ULs) (Fig. 2E). Interestingly, UL128 binds the same site on gH/gL as gO, forming a disulfide with gL-144-Cys (116). UL130 and UL131A do not bind gH or gL directly (28, 29, 140). All three ULs are small, soluble, and contain very few glycosylation sites. UL128 and UL131A both migrate at approximately 20 kDa, and the third component, UL130, is slightly larger at ~ 35 kDa (116). The first structure of the pentamer, bound to human nAbs, was reported in 2018 (141). Since then, numerous other studies have reported structures of the pentamer bound to nAbs or HCMV receptors such as Neuropilin-2 (Nrp2) and Thrombomodulin (THBD) (28, 29, 140).

Functionally, the PC is required for entry into epithelial, endothelial, monocytic, and a subset of dendritic cells (112, 142–144). It is, however, not required for entry into fibroblasts (136). In fact, some strains and mutant viruses lacking a functional PC, such as AD169, are even more adept at infecting fibroblasts (136, 145). Unlike the trimer, which promotes a pH-independent mechanism of entry, the pentamer has been shown to promote HCMV entry through pH-dependent endocytosis (145, 146). A comprehensive description of the PC, including its structure, functions, and additional characteristics that are outside the scope of this report, can be found in the review by Malito et al. (147).

### Overview of the anti-pentamer antibodies

MAbs against pentamer are relatively new as compared to those against other HCMV antigens, with the first pentamer-specific mAbs reported in 2005 (112) (Table S4). As with gH/gL, the first landmark study of pentamer-specific mAbs was conducted by Macagno et al. in 2010 (94). They not only introduced 16 additional nAbs to the field (Table S4) but also demonstrated the remarkable potency of these nAbs. nAbs specific to the ULs exhibited IC_90_s that were 100-fold to 1,000-fold lower than those previously reported for any other HCMV antigen, including those against gH/gL. Additionally, these nAbs neutralized infection only in epithelial cells but not in fibroblasts, which have been the standard for viral neutralization assays and remain so to this day; ~21.2% of all mAbs against gH/gL-complexes are specific to the pentamer ULs (Fig. 2B), and most of these mAbs are neutralizing (Fig. 2C, left). Two such nAbs, 15D8 and 8I21, have since been used extensively as controls in other HCMV studies (28, 29, 116, 140).

### Clinical cocktail: CSJ148

The generation of such potent nAbs led to the development of two clinical mAb cocktails, both of which included pentamer-specific components. One such cocktail, CSJ148, composed of nAbs LJP538 and LJP539 that targeted gB and pentamer, respectively, was used as a potential prophylactic in hematopoietic cell transplant recipients (148). LJP539, designated as 4I22 in the Macagno 2010 study (93, 94), is an IgG1 that was not neutralizing in fibroblasts but had a remarkably low IC_90_ of 0.0015 μg/mL in ARPE-19 epithelial cells. Its epitope was mapped to a site overlapping UL130 and UL131A, but its precise epitope has not yet been determined (93, 94). *In vitro*, LJP539 potently inhibited infection of two lab-adapted strains of HCMV as well as clinical isolates in multiple epithelial and endothelial cell lines (93). In contrast, LJP538, designated 7H3 in the 2010 study (93, 94), neutralized epithelial, endothelial cells, and fibroblasts yet had IC_90_s, on average, >100-fold higher than those of LJP539 (93). Despite robust *in vitro* results (93) and no adverse effects in Phase 1 (149), the cocktail did not meet the primary efficacy endpoints in Phase 2 (148). It is possible that this was due to a lack of effector functions, given that only LJP538 but not LJP539 showed minimal levels of ADCC activation *in vitro* (93).

### Clinical cocktail: RG7667

Antibody cocktail RG7667, composed of nAbs MCMV5322A and MCMV3068A, was used in a Phase 2 clinical study as a potential prophylactic for kidney transplant recipients (129, 150). The first of these mAbs, MCMV5322A (128), was derived from MSL-109, which was discussed in detail earlier. Although its epitope had not yet been mapped, MCMV3068A was reported as being specific to pentamer, although the data demonstrating this appear to be proprietary information (129, 150). RG7667 reduced HCMV viremia at 24 weeks post-transplant but did not meet the endpoint criteria at 12 weeks post-transplant and, thus, did not advance to Phase 3 (150).

ADCC has been associated with decreased risk of congenital HCMV transmission (38). Some, but not all, antibody components of clinical cocktails have shown ADCC-activation *in vitro*. In the case of antibody cocktail CSJ148, LJP538, but not LJP539, showed minimal levels of ADCC *in vitro* (93). In the case of antibody cocktail RG7667, neither nAb demonstrated detectable levels of ADCC or complement-dependent cytotoxicity *in vitro* (150). It is difficult to evaluate whether the effector functions of specific mAbs correlate with *in vivo* disease transmission in transplant patients because all study participants typically receive some form of immunosuppressive anti-rejection drug. However, it has been shown that T-cell responses are critical for long-term protection against HCMV in solid-organ transplant patients (8).

Despite their robust neutralizing properties, pentamer-specific nAbs have not yet been successful as prophylactics in transplant recipients. It remains unclear whether the presence of any pentamer-specific nAbs correlates with intrauterine transmission, as recent studies have yielded conflicting results (151, 152). NAbs that additionally possess effector functions may prove to be more useful in *in vivo* studies.

### mRNA-1647 vaccine candidate

Antibodies against ULs exhibit the highest potency of any anti-HCMV nAbs published to date (see *Overview of Anti-Pentamer Antibodies*) (Table S4). This phenotype makes the pentamer an attractive vaccine candidate. It is, therefore, not surprising that mRNA-1647, composed of mRNAs targeting gB and pentamer, was the first HCMV vaccine candidate to enter Phase 3 clinical trials (153–155). Notably, the vaccine demonstrated broad neutralization across 13 HCMV strains, and in multiple cell types, anti-pentamer IgG was the predominant nAb in seronegative individuals (155). Additionally, the UL-specific mAbs elicited stronger T-cell responses in seronegative individuals (153, 155). Seropositive participants also showed a higher baseline response to gB and pentamer, further validating the importance of these two antigens (155). However, the ADCP responses were lower than those observed with a previous vaccine candidate, gB/MF59 (156). This is especially striking given that ADCC responses were higher in mRNA-1647 than in gB/MF59 (156). Although mRNA-1647 was ultimately unsuccessful in Phase 3, further analyses of Phase 3 samples will provide invaluable information to identify correlates of protection against congenital HCMV. These insights and the development of this mRNA vaccine candidate would not be possible without the original studies documenting the potency of pentamer-specific nAbs.

### The GATE glycoprotein complex

In 2016, gH was, for the first time, found to bind a protein other than gL (117). This glycoprotein, UL116, was found to compete with gL for gH binding. Most recently, in 2025, the GATE glycoprotein complex—composed of gH, UL116, and UL141—was identified. UL141 is, perhaps, the most peculiar member of the GATE complex. In 2021, Vlahava et al. reported antibodies against nonstructural viral antigens that robustly induced ADCC (39). One of their targets was the immunoevasin UL141 (157), and anti-UL141 mAbs induced a robust NK cell response when expressed in isolation and as a pool, with a much higher response produced by the pool. Unfortunately, individual clone names for these mAbs were not published. The 2025 study showed that UL141 within the GATE complex enhances HCMV infection in fibroblasts and endothelial cells and may act as the receptor-binding protein of the GATE complex, similarly to gO in the trimer (118). Taken together, these studies position the GATE complex as a fascinating new target for mAb therapeutics, given that mAbs against gH and UL141 have already shown robust neutralization and effector functions, respectively.

## gM/gN

### gM/gN structure and function

**Fig 3:**
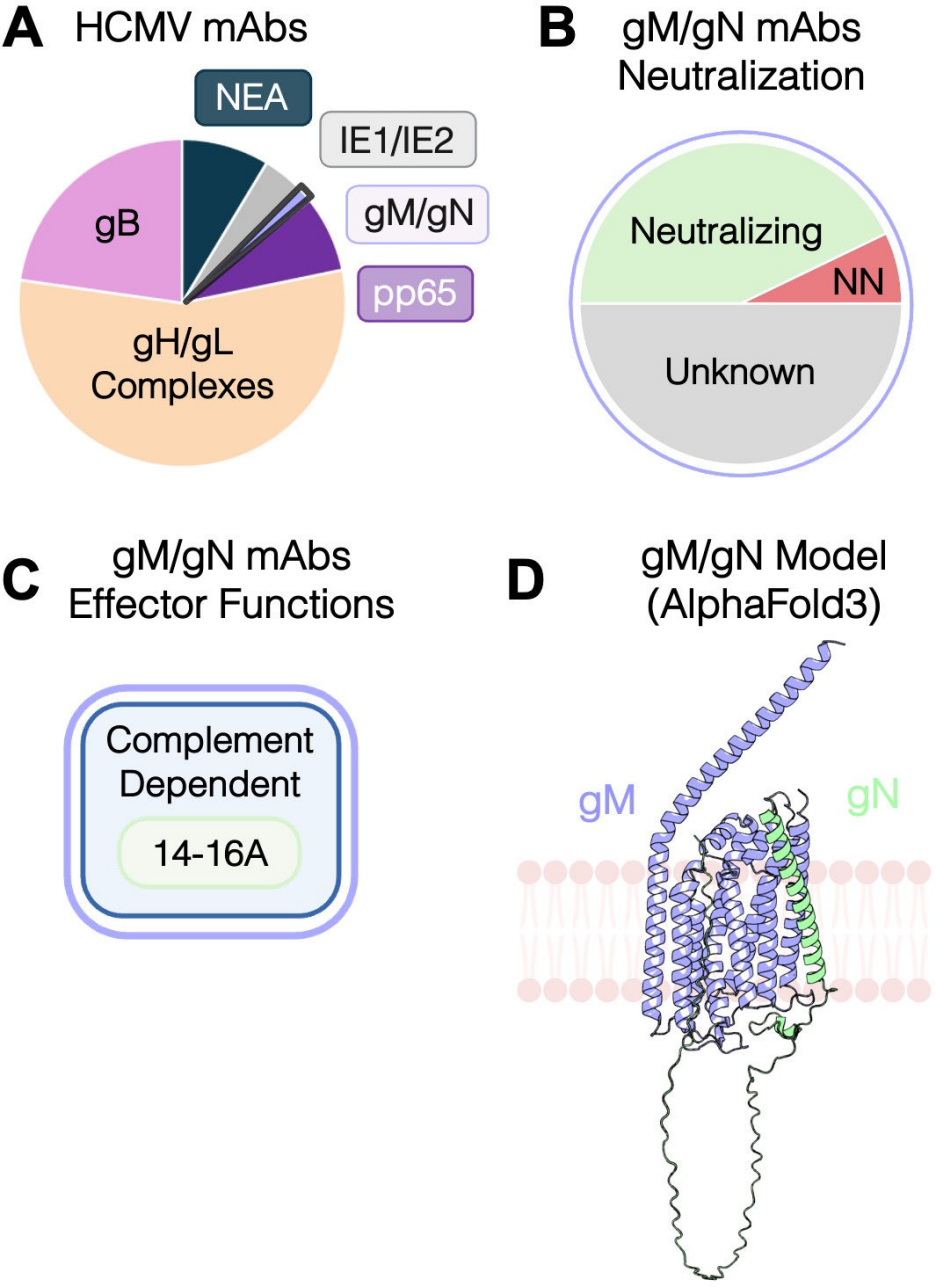
Few isolated mAbs target the gM/gN complex. (**A**) Pie chart representing the percentage of all HCMV mAbs that have been isolated. Only 1.6% of these target gM/gN. Non-envelope antigens abbreviated as “NEA.” (**B**) All gM/gN mAbs are further grouped by their neutralization properties: neutralizing mAbs (6/14, 42.9%); non-neutralizing (NN, 1/14, 7.1%); and mAbs with unknown neutralization properties (7/14, 50%). (**C**) Only one gM/gN mAb clone has been reported to have an effector function (complement-dependent neutralization). (**D**) AlphaFold3 model of AD169 gM/gN (ipTM = 0.59, pTM = 0.63). For detailed information on individual mAb clones, see Table S5.

The gM/gN glycoprotein complex, historically referred to as gC-II (158–162), is the most abundant structural component of the HCMV envelope (44), although a few mAbs have been isolated against this complex (Fig. 3A through C).

gM, the larger component of the heterodimer, is a ~45 kDa, type III membrane protein encoded by the UL100 gene (158, 159, 163) (Fig. 3D). This gene is conserved across many members of the *Orthoherpesviridae* family, including HSV-1, pseudorabies virus (PRV), and EBV (164–166). Functionally, gM has been reported to be involved in viral entry and cell-to-cell spread, although it is nonessential for *in vitro* replication in most *Orthoherpesviridae* (164, 167–169). It is, however, important for the *in vivo* replication of PRV and HSV-1 (170, 171) in animal models of infection and is essential for productive *in vitro* replication of HCMV in fibroblasts (159, 163, 172).

The second component of this complex, gN, is a ~40–50 kDa, type I membrane protein encoded by the UL73 gene (158, 159, 163) (Fig. 3D) and extensively N- and O-linked glycosylated (158, 159, 162, 163, 173). Unlike that of gM, the gN sequence varies extensively among strains of HCMV (139, 163, 173–175), potentially suggesting immune-driven evolution (111, 176). As such, gN has been classified into four main genotypes, with two, gN-3 and gN-4, having two subgroups each (163, 177). These genotypes can alter the neutralizing Ab response to the whole virus, as shown by significant differences in bulk anti-HCMV nAb titers, including responses targeting gN, gB, and gH, when AD169 variants differing only in gN genotype were compared (178, 179). Additionally, the C-terminus of gN is important for secondary envelopment and viral replication of HCMV (160, 163, 180). Structures of this complex or its individual components have not yet been determined (Fig. 3D).

Natural HCMV infection elicits an Ab response against the gM/gN complex, although the nAb titers are lower than those against gB (5). This may, in part, be due to the role of gN in immune evasion. As it is the most abundant glycoprotein complex on the virion surface (44), its heavy glycosylation is thought to shield a large portion of the virion surface from immune recognition (176). Despite its abundance and potentially pivotal role in the HCMV immune response, it remains one of the least characterized glycoprotein complexes on the HCMV surface (Fig. 3A).

### Murine anti-gM/gN antibodies

MAbs against gM/gN are among the earliest to be described and isolated against HCMV (Table S5). In 1985, murine mAbs 14-9 and 14-16A were reported by Britt and Auger to bind to gM/gN—then referred to as gp62 or gp65 (158, 162)—and neutralize AD169, Towne, and Coff strains of HCMV (162). Curiously, 14-16A is one of the few documented anti-HCMV nAbs that is entirely complement-dependent (163, 176) (Fig. 3C). Since then, these mAbs, specifically 14-16A, have been used extensively in downstream functional studies and as controls for antibody isolation (158, 163, 172, 180). However, the specific epitopes of either mAb have not yet been determined.

The next generation of mAbs against gM/gN was isolated and characterized in the 1990s by Kari et al. (160, 161, 181–183) (Table S5). The respective antigens of these murine mAbs were successfully identified and described based on their ability to immunoprecipitate either gM or gN (161, 183). Of this group, mAb 9E10 has been characterized most extensively and shown to neutralize both the Towne and Toledo strains of HCMV in the absence of complement (182). The neutralization properties of the rest of the anti-gM/gN mAbs isolated alongside 9E10 have not yet been characterized (Fig. 3B).

### Human anti-gM/gN antibodies

Most recently, Shimamura et al. described the first human gM/gN mAbs and demonstrated their ability to potently neutralize AD169, Toledo, and TR strains, both in the presence and absence of complement (163). While this study was crucial for advancing the understanding of gM/gN Ab responses, information on the individual mAbs is unavailable because they were pooled before use.

## NON-ENVELOPE ANTIGENS

### IE1/IE2

Several non-envelope antigens, notably IE1 and IE2, have been targeted by mAbs (Table S6). Immediate Early (IE) genes are the first to be expressed during HCMV lytic infection, and IE1, IE2, and IE55 share their first 85 N-terminal residues (13). IE1, a 72 kDa protein, historically referred to as pp72 or pp68 (52, 121, 162), is encoded by the UL123 gene (13). It activates transcription, regulates intrinsic and innate responses to infection, and is required for replication at lower multiplicities of infection (13). IE2, encoded by the UL122 gene (13), is an 86-kDa protein considered the most important HCMV regulatory protein (184, 185). It is the strongest known transcriptional regulator of both viral and cellular gene expression (13, 185), even binding to its own promoter during the early and late phases of infection to prevent transcription and repress IE expression (13). Both proteins have been implicated in regulating the antiviral immune response. IE1 can indirectly downregulate the response to IFN through STAT1 and 2, whereas IE2 represses transcription and induces the degradation of immature IL-1β (13).

Despite the shared 85 residues, all known IE mAbs are specific to IE1, with one, E13, binding to a region shared between IE1 and IE2 (Table S6). However, the epitopes of these mAbs have not yet been characterized, and IE1, IE2, and a third IE, IE55, share the first 85 N-terminal residues (13). Some of the mAbs listed as being specific to IE1 may also bind IE2 or IE55, especially since a substantial portion were discovered during the early stages of HCMV studies (Table S6) when epitope mapping was significantly more challenging. However, it is also likely that the essential role of IE2 in HCMV infection and immune evasion has, in turn, exerted evolutionary pressure to shield IE2, thereby minimizing Ab recognition without disrupting viral replication.

### pp65 (UL83)

A significant portion of mAbs isolated against non-enveloped antigens target pp65, a tegument protein encoded by UL83 (Table S7). As the most abundant HCMV tegument protein, it has been implicated in both mature virus particle formation and in modulating the host immune response. While it is not essential for AD169 replication in fibroblasts *in vitro*, it is required for efficient viral growth in macrophages (186). It is thought to interact with IFI16, targeting it to drive IE2 gene expression and limit host interferon responses (187). A pp65 deletion mutant results in delayed IE86 expression, a gene product of IE2, response, and therefore, a delay in downmodulation of beta interferon (187, 188). This was associated with a decrease in pp71, another major tegument protein, suggesting that pp65 and pp71 may interact (188). Finally, pp65 has been shown to assist in the incorporation of two additional tegument proteins, pUL69 and pUL97, into the viral particle (186).

While there are nearly 70 anti-pp65 mAbs published to date (Table S7), the majority of studies have utilized a commercialized variant of the CH12 mAb, first published in 1982 (189). Although there are two published anti-pp65 nAbs, CH65 and CH134, pp65 mAbs have not been associated with any clinical studies. These mAbs are instead used as markers of mature virus particles in microscopy and protein interaction assays.

### Other non-envelope antigens

In addition to the proteins described above, tegument proteins UL48, UL48/49, and UL99, as well as a variety of other non-glycoprotein targets, have been the subject of HCMV Ab studies (67, 121, 180, 190–192). These mAbs are largely murine, with most isolated in the 1980s (Table S8). Notably, mAb 28-4, which targets the major capsid protein, was used as a control in the 2008 study that characterized the binding interactions between the pentamer ULs and gH/gL (145). While none of these mAbs have been used in any clinical studies, they are essential for investigating HCMV biology. Some, particularly those from 1984 to 1989, should be revisited to identify their antigens conclusively.

## CONCLUDING REMARKS

### gB

Several clear trends have emerged from the accumulated body of research on HCMV mAbs. Given the number of mAbs summarized in this report (Tables S1 to S8), the field now has sufficient reagents to answer a variety of outstanding questions, with a few exceptions. Prior to a recent 2026 study, there were no mAbs that exhibited a conformational preference for prefusion gB (Fig. 1B), although a prefusion-like gB (gB-C7) (95) had been reported to elicit a modest neutralizing Ab response (193). However, a study by McClave et al. identified a mAb, MLCB1, that showed an affinity preference for prefusion over intermediate and postfusion gB (91). Given the success of prefusion-specific mAbs against RSV F (31), it was not surprising that this mAb showed robust neutralizing properties. However, the neutralization was, in some cases, on par with mAbs displaying a preference for other conformations of gB. It is, therefore, possible that conformational specificity in HCMV gB may not be as strong a predictor of neutralization potency as it is for RSV. This is further supported by two studies demonstrating that immunization with prefusion gB constructs did not elicit stronger nAb responses but was, instead, comparable to those elicited by postfusion gB (91, 193). Beyond neutralization, comparison of conformation-specific mAbs may provide insight into the intermediate gB conformation, shedding further light on the viral fusion mechanism. Characterization of mAbs against the newly defined AD-6 may yield similar insights because AD-6 is predicted to be exposed in early intermediates(97) (Fig. 1C through E).

### gH/gL and complexes

Although conclusions cannot be drawn regarding trimer mAbs due to their limited quantity (Fig. 2B), pentamer-specific mAbs are consistently the most potent neutralizers, often outperforming gB- and gH/gL-directed antibodies by orders of magnitude in epithelial and endothelial cell neutralization assays (29, 94). However, many of the most potent neutralizers do not function in fibroblasts (94), suggesting that they may need to be combined with mAbs targeting other antigens to create a more protective cocktail. Recent attempts have been made to divide the pentamer into antigenic regions, similarly to the antigenic domains of gB (29). This additional nomenclature would enable the field to map the immunogenicity of each region more precisely. Working with the pentamer can be technically challenging; hence, identifying smaller antigenic regions may enable work with recombinant peptides. This peptide-based approach, utilized with recombinant gB AD-5 (90), shows promise and offers improvements over research with whole proteins.

### gM/gN

Future studies should leverage the available pool of Abs to structurally characterize the gM/gN complex and its components. Structural work on gB and the gH/gL complexes has advanced the classification of their respective Abs, leading to a better understanding of HCMV immunogenicity (28, 29, 56, 95, 133, 140, 141). Similar work should be done to map the epitopes of the available anti-gM/gN mAbs. Additionally, it has been hypothesized that extensive modification by glycans and genetic polymorphism contribute to the mechanism by which gN modulates nAb evasion (163, 176). High-resolution structures may provide insight into these questions by mapping sites of high sequence variation and heavy glycosylation. Finally, the identification and characterization of individual human mAbs against gM/gN would broaden the available resources for studying this abundant yet underexplored glycoprotein complex.

### State of the field

The field of anti-HCMV antibodies has been primarily driven forward by the isolation of new clones, leaving the epitopes of many historical mAbs unmapped and their functions under-characterized. For example, a few studies have tested the effector functions of any mAbs mentioned in this report. Some clones have been characterized for complement-dependent neutralization (61, 67, 82, 182), but, to our knowledge, functions such as ADCC and ADCP have been studied primarily in whole sera (38, 40, 102, 155, 156). One notable exception is a study by Vlahava et al. that specifically screened for viral antigens successfully activating ADCC, finding that most of these were non-structural proteins (39). Relying exclusively on neutralization as a predictor of clinical protection may explain why, to date, no marketable anti-HCMV clinical mAb cocktails have been developed.

More recent studies identified a role for ADCC and ADCP in protection from HCMV transmission, but only in the case of cCMV transmission (38–40), suggesting that the correlates of protection for post-transplant HCMV transmission and cCMV may differ. In the case of cCMV, ADCC-, and ADCP-activating antibodies may be promising candidates for inclusion in downstream clinical antibody cocktails.

The field of Ebola faced a similar problem before the development of the Inmazeb cocktail in the 2010s (33, 194). The components of this cocktail were identified through immunization of VelocImmune mice, which use human immunoglobulin variable regions, with Ebola virus glycoprotein (GP). Unfortunately, because HCMV encodes multiple surface glycoproteins, the field must first identify an appropriate antigen for use in similar studies. Studies in HCMV are also limited by a lack of high-throughput animal models, due to the narrow host tropism of HCMV.

However, much can still be gleaned from the Ebola field. In 2018, the field conducted an extensive study of all published mAbs targeting Ebola GP to identify correlates of protection (35). This study identified ADCC activation as a strong correlate of protection, in addition to neutralization. Given that ADCC and ADCP have been shown to play roles in protection against HCMV (38, 40), a similar set of studies on HCMV would be warranted and might yield similar insights. Nonetheless, reclassifying previously identified mAbs based on their effector functions would serve as an excellent starting point.

## SUPPLEMENTAL MATERIAL

Supplemental tables jvi.00325-26-s0001.xlsx Tables S1 to S8.

ASM does not own the copyrights to Supplemental Material that may be linked to, or accessed through, an article. The authors have granted ASM a non-exclusive, world-wide license to publish the Supplemental Material files. Please contact the corresponding author directly for reuse.
